# Endoscopic total parathyroidectomy via anterior chest approach with forearm autotransplantation for secondary hyperparathyroidism: a comparison of surgical results with open total parathyroidectomy with autotransplantation

**DOI:** 10.3389/fonc.2023.1137278

**Published:** 2023-05-01

**Authors:** Tebo Hua, Jinfeng Lou, Ye Zhu, Yong Luo, Hai Zhang, Jiahui Yang

**Affiliations:** ^1^ Department of Thyroid Breast Surgery, Ningbo Medical Centre Lihuili Hospital, Ningbo, Zhejiang, China; ^2^ Department of Anesthesiology, Ningbo Medical Centre Lihuili Hospital, Ningbo, Zhejiang, China

**Keywords:** endoscopic, hyperparathyroidism, parathyroid, parathyroidectomy, surgery

## Abstract

**Objective:**

This paper aimed to evaluate the clinical value of performing an endoscopic total parathyroidectomy through anterior chest approach with autotransplantation (EACtPTx+AT) in treating secondary hyperparathyroidism (SHPT) to summarize and share the clinical experience.

**Methods:**

24 patients with SHPT were retrospectively analyzed:11 patients underwent open total parathyroidectomy with autotransplantation (OtPTx+AT Group) and 13 patients underwent endoscopic parathyroidectomy through anterior chest approach with autotransplantation (EACtPTx+AT Group). Comparing the two groups regarding the following factors: (1) operating conditions, such as the blood loss during the operation, the length of time spent on the operating table, the number of parathyroid glands removed, postoperative drainage volume and hospital stay. (2) clinical efficacy, parathyroid hormone (PTH) and serum calcium (Ca) levels. (3) postoperative complications.

**Results:**

First, there were no significant differences in the number of parathyroid gland resection, operation time, intraoperative blood loss and hospital stay between the two groups. While there were significant differences in postoperative drainage volume between the two groups. Second, the two groups preoperative PTH and preoperative serum calcium decreased significantly compared with those of the two groups after surgery and there was a statistically significant difference. Thirdly, there was no postoperative bleeding, hoarseness or choking in the two groups and no conversion to open surgery case in EACtPTx+AT group.

**Conclusion:**

Endoscopic treatment of SHPT using the anterior chest approach with forearm autotransplantation significantly improves clinical symptoms and lowers levels of PTH and serum calcium after the operation. The results confirm the operation’s safety and effectiveness.

## Introduction

Secondary hyperparathyroidism (SHPT) is one of the most commonly seen complication in patients with chronic renal failure (CRF), with a high incidence worldwide and is often referred to as renal hyperparathyroidism. It is found that surgical intervention is the best option when other medical treatments did not work by relieving their symptoms and reducing mortality (1). There are a number of surgical interventions that can be used to treat SHPT but total parathyroidectomy with autotransplantation of the forearm is the most effective treatment (2, 3).

The initial approach of surgical intervention to treat secondary hyperparathyroidism was performed total parathyroidectomy through a central cervical access and autotransplantation at forearm. In recent years, thyroid and parathyroid surgeries have been attempted endoscopically through various approaches with the development of laparoscopic and endoscopic surgery. The indications for endoscopic thyroid or parathyroid surgery (ETS/EPTS) have been further expanded and gradually popularized in clinical practice and ETS/EPTS with different approaches have been reported successively. In 1996 Gagner introduced endoscopic parathyroidectomy for the first time (4), the endoscopic surgery of thyroid or parathyroid glands has been performed in a variety of ways, such as through the axillary system, breast, or anterior chest region. The anterior chest approach is the preferred external cervical approach for scarless endoscopic thyroidectomy (SET) of the neck. Asanakietkul’s team successfully performed the transoral or transvestibular approach in 12 patients with primary and renal hyperparathyroidism in 2016. The approach gained acceptance, using one 10 mm and two 5 mm incisions in the lower lip vestibule (5). According to their research, it is a safe and feasible treatment, particularly for patients who are concerned about their appearance. Kitano et al. and Ikeda et al. performed endoscopic parathyroidectomy by anterior chest approach, using mechanical retractors to create adequate working spaces in the former group and carbon dioxide inhalation for the latter group (6, 7). It provides the best cosmetic results in comparison to conventional methods. However, with the extensive development of endoscopic technology in the field of thyroid surgery, there are many controversies in the academic field.

The researcher found very few surgeons using anterior chest approach endoscopy in secondary parathyroid surgery and it was believed that total parathyroidectomy through this approach is more beneficial for finding and identifying diseased parathyroid glands and it is also good news for patients with cosmetic needs. So, our center used endoscopic total parathyroidectomy via anterior chest approach with forearm autotransplantation for secondary hyperparathyroidism patients and found that the therapeutic effect was good with no scar on the neck and minimally invasive cosmetic effect. These clinical data were retrospectively analyzed and shared our treatment experience and surgical effects.

## Patients and methods

### Patients

We analyzed all of the surgical procedures performed total parathyroidectomy with endoscopic total parathyroidectomy through anterior chest approach with autotransplantation (EACtPTx+AT Group) in 13 patients with SHPT and the control group consisted of 11 patients with SHPT who underwent open cervical approach (OtPTx+AT Group) from January 2016 to August 2022. Inclusion Criteria: secondary hyperparathyroidism in the kidney with parathyroid hormone levels greater than 800 pg/mL, or serum calcium levels greater than 10 mg/dL or clinical symptoms. Exclusion criteria: 1. Primary hyperparathyroidism, 2. Having a history of significant thyroiditis. 3. Having undergone neck surgery or irradiation in the past. 4. Suspecting malignancy. Hemodialysis was continuously provided to all patients throughout this period. All SHPT patients routinely underwent hemodialysis treatments. In accordance with the Declaration of Helsinki, this study was approved by our hospital’s Ethics Committee (Approval NO.KY2022SL413-01).

### Preoperative procedures

**Figure 1:**
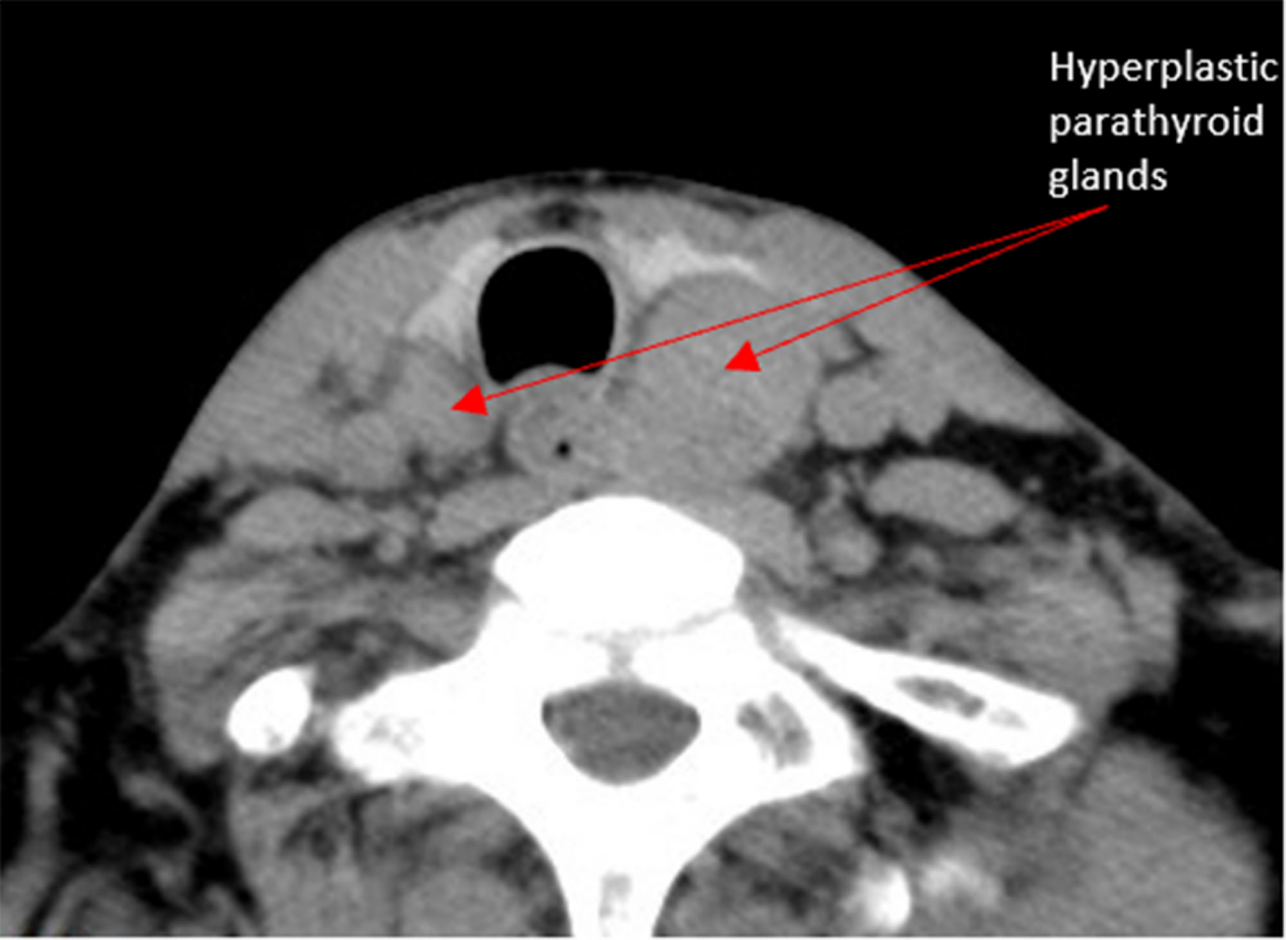
CT scan of the neck suggests parathyroid hyperplasia.

The timing of surgery at the dialysis interval, adjust the patient’s dialysis schedule and dialysis contents according to the surgical plan, no heparin dialysis was performed 24 hours before surgery. The preoperative location of the parathyroid gland was determined by color ultrasound and CT (**Figure 1**).

### Surgical technique

**Figure 2:**
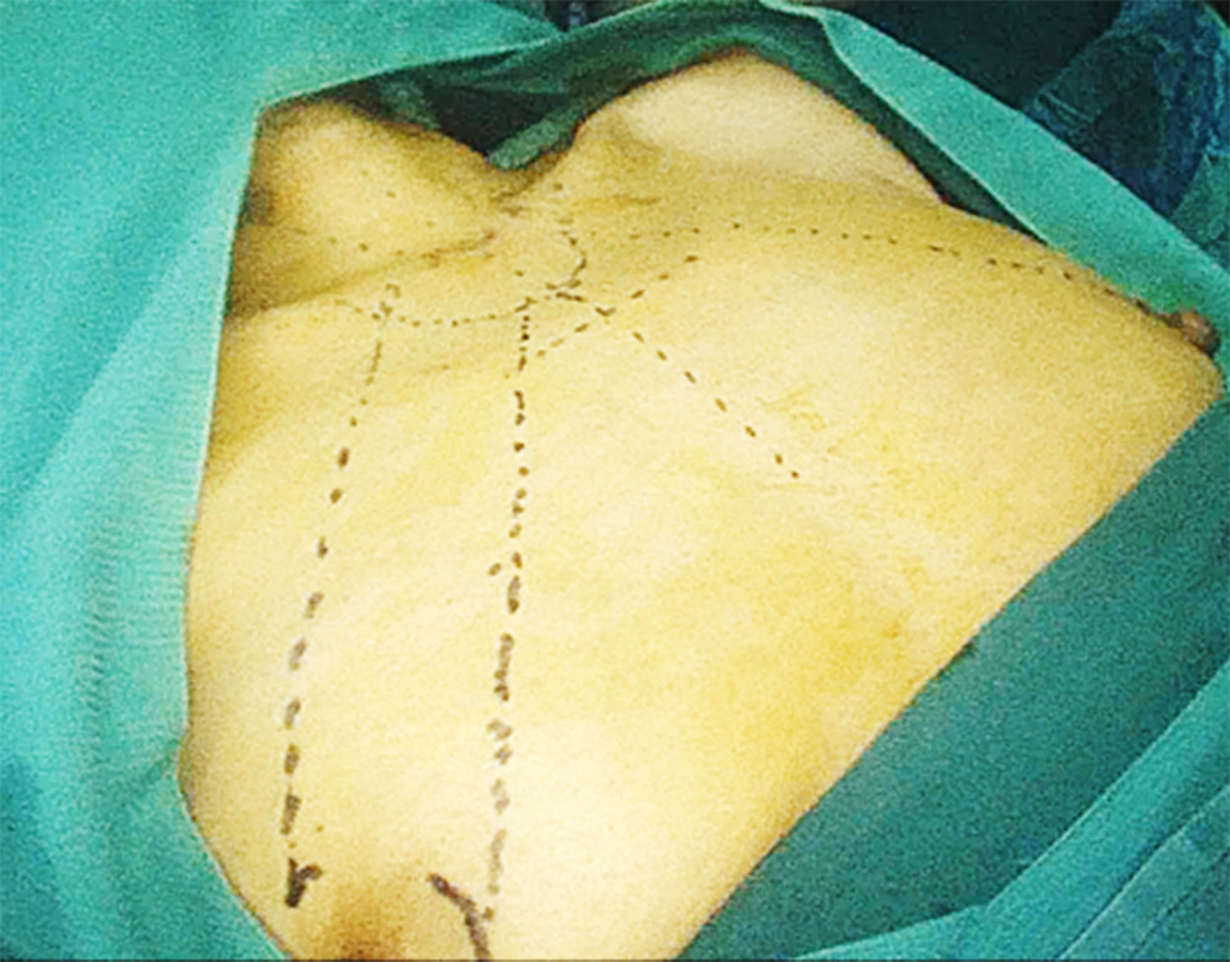
Three Choice of incisions. For female a 10 mm incision is made at 3 o’clock on the right areola and a 5 mm incision is made at 12 o’clock on the right areola and 11 o’clock on the left areola.

**Figure 3:**
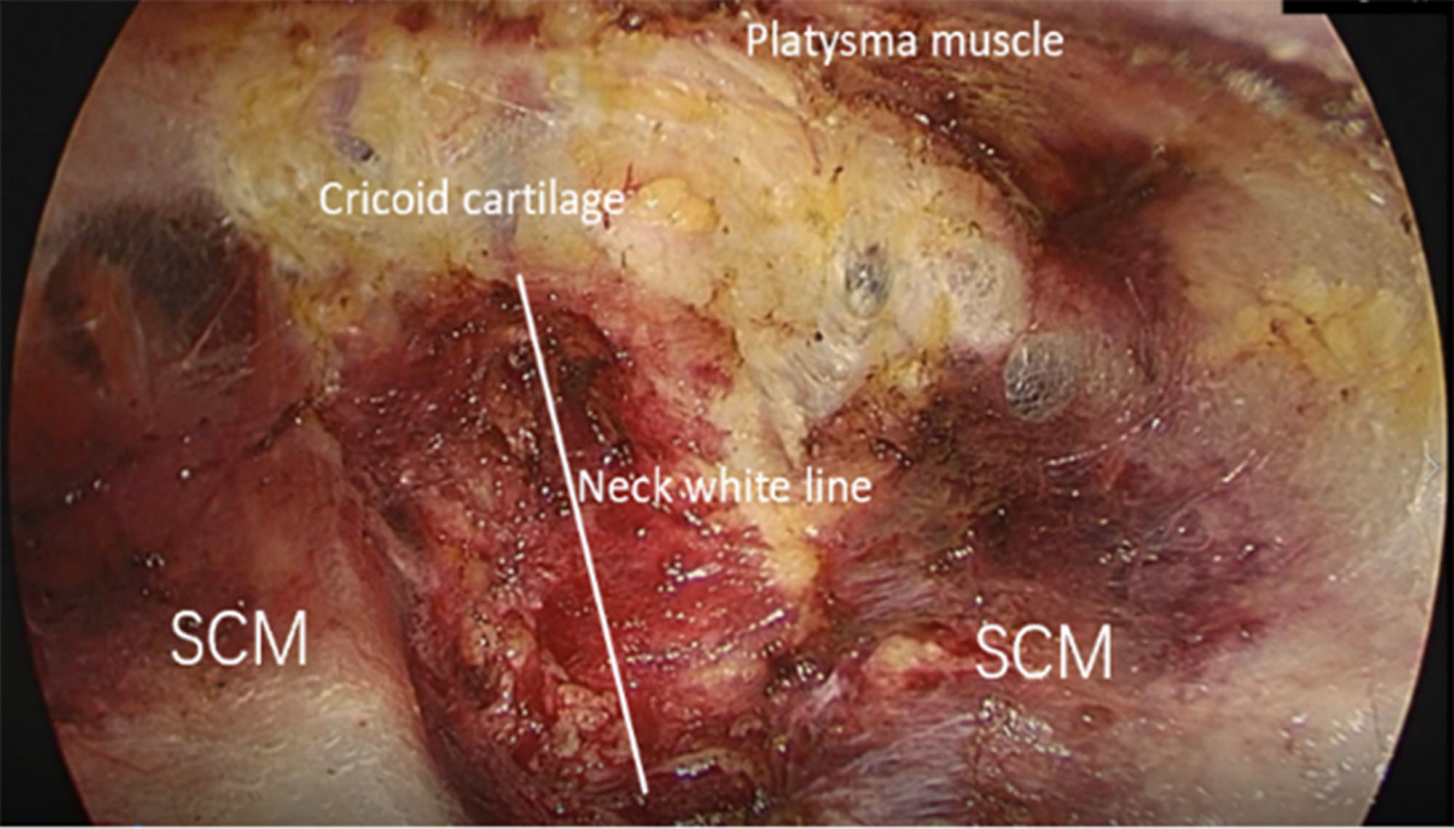
Operation space establishment and the structure of dissection under endoscope.

**Figure 4:**
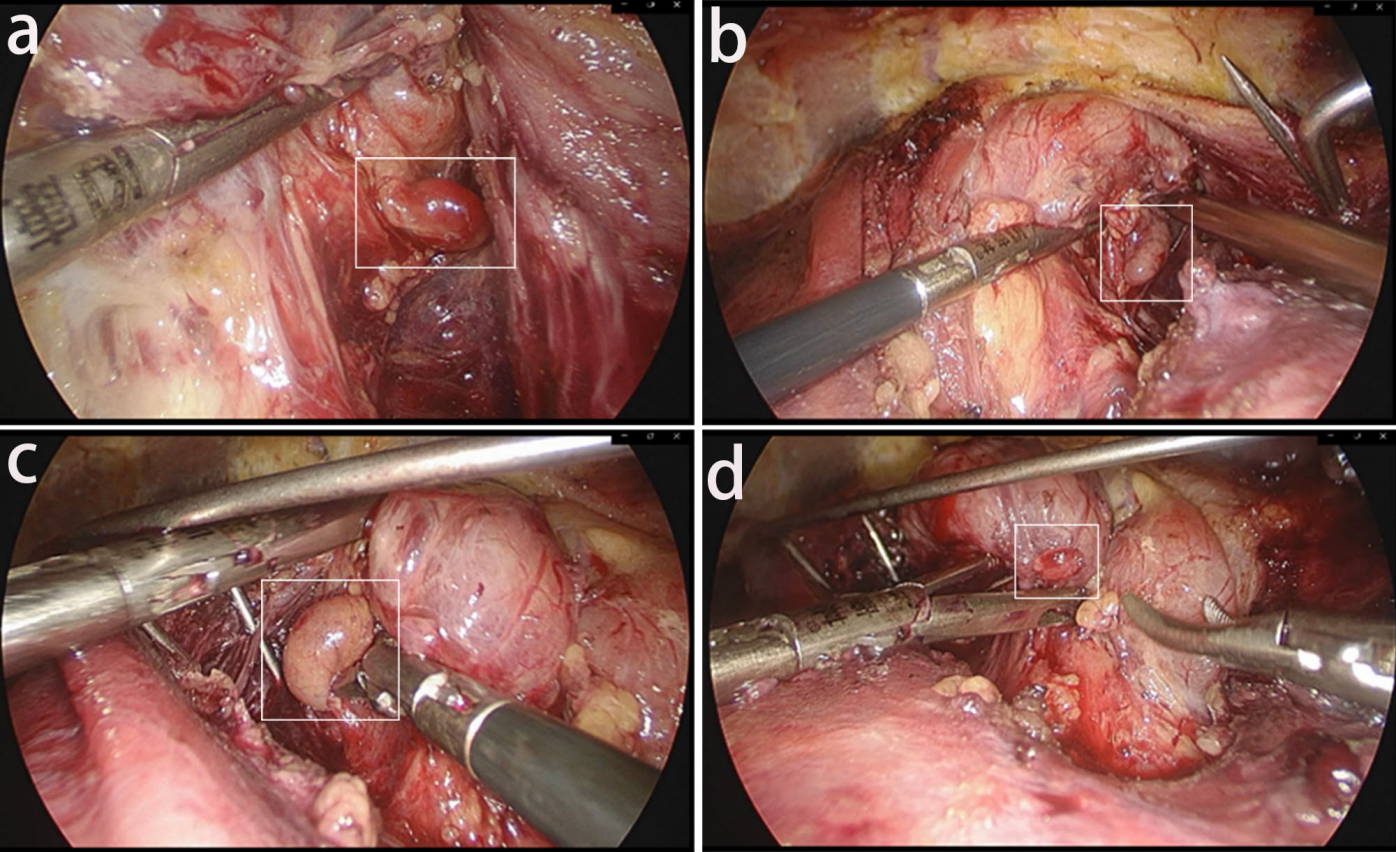
The total endoscopic exposure of the four parathyroids: The posterior surface of the thyroid was explored to search for the parathyroid glands. **(A)** Left upper parathyroid gland. **(B)** Left lower parathyroid gland. **(C)** Right upper parathyroid gland. **(D)** Right lower parathyroid gland.

1. Incision selection, the open surgical incision can be adjusted according to the patient’s body shape and preoperative positioning, usually a mid-neck and low collar incision is chosen. The anterior chest approach was chosen for endoscopic surgery, If the patient is a female, choose the whole areola approach, a 10 mm incision is made at 3 o’clock on the right areola and a 5 mm incision is made at 12 o’clock on the right areola and 11 o’clock on the left areola (**Figure 2**). If the patient is male, a 10 mm incision is made from the right parasternal to the fourth intercostal space and a 5 mm incision is made at the third intercostal space, oriented at 1 o’clock on the right breast and 11 o’clock on the left breast.

2. The operation to prepare 13 patients were prepared for endoscopic parathyroidectomy under general anesthesia. After the patients were placed in the supine position, a pillow was placed under the shoulders to lengthen the head and neck. The patient’s bilateral lower extremities were abducted at an angle (45-60°C) and properly secured with bandages. The arms are inwardly folded at the sides of the body and secured. The operator stands between the patient’s legs, the range assistant stands on the patient’s right side, the first assistant stands on the patient’s left side, and the monitor is placed on the patient’s head.

3. Space building the use of special equipment for endoscopic thyroid, including visualized, small-headed, extended trocar and detachment rods, multifunctional detachment forceps for nerve monitoring, minilap and special pulling hooks, can effectively shorten the operation time and reduce surgical complications. The middle cut for the observation hole, both sides of the cut for the operation hole, and choose the extension trocar. To facilitate the separation of tissue gaps and reduce bleeding, use 80 mL of saline containing 1:100,000 epinephrine mixed with 20 mL of ropivacaine. An injection of the tumescent solution was administered along the surgical path in the subcutaneous layer of the anterior chest, injections are limited to the anterior chest wall. Initially, the flap is bluntly peeled with a separating rod. In the anterior thoracic wall, the separation is between the deep fascia and the pectoralis major fascia as far as possible; in the neck, the separation is between the superficial cervical fascia and the superficial deep cervical fascia as far as possible. After blunt dissection, an endoscopic thyroid-specific puncture device is inserted through each incision. Carbon dioxide gas is injected at a pressure of 6 mm Hg. The flap continued to be separated, expanding the operative space to the level of the thyroid cartilage on both sides and laterally to the sternocleidomastoid muscle (**Figure 3**).

4. Gland resection, After the surgical maneuvering space is established, a midline dissection is made from the thyroid cartilage to the cervical white line between the strap muscles of the sternotomy. The anterior cervical muscles are pulled to the sides by using endoscopic thyroid pulling hooks; the lateral thyroid gland is separated and turned up to the opposite side; if necessary, the retracting vessels of the thyroid gland are cut during the exposure; with the surgical field completely exposed, all parathyroid glands are explored and lesions are removed according to CT and ultrasound findings and parathyroid anatomy, with care taken to protect the recurrent laryngeal nerve during the removal process; the parathyroid lesion tissue is sent for intraoperative The parathyroid tissue was sent for frozen section, and the remaining tissue was placed in normal saline for transplantation. If less than 4 parathyroid glands are found intraoperatively and iPTH ≥ 400 ng/L is measured after intraoperative removal of “all parathyroid glands”, ectopic parathyroid glands should be fully explored in addition to the normal anatomic location of the parathyroid glands. Attention should be paid to common sites of ectopic parathyroid glands, such as the thymus, perioesophagus, carotid sheath, and anterior mediastinum. In this study, there were no ectopic parathyroid cases in the enrolled patients (**Figure 4**).

5. Intraoperative evaluation, Intraoperative parathyroid hormone (iPTH) levels are measured by sampling the patient’s blood 20 minutes after removal of all parathyroid glands. It is now clinically accepted that an 80% decrease in iPTH measured 20 minutes after PTx compared to preoperative levels is sufficient to confirm that the parathyroid glands have been completely removed.

6. Autotransplantation, Participants in the intervention group underwent forearm autograft surgery. The medical team preserved 30-60 mg of the smallest non-nodular hyperplastic parathyroid tissue, cut it to a size of 1 mm × 1 mm × 1 mm, and implanted it subcutaneously into the radial forearm to create one or more “muscle capsules” without hematoma formation, in which small pieces of parathyroid tissue were placed in full contact with the muscle. The team marks the graft site with non-absorbable sutures and closes the subcutaneous tissue and skin after hemostasis.

### Postoperative management

The medical team recommended that the patient be placed in a semi-recumbent position upon awakening. Postoperative management of the patient included (1) continuous observation of the patient’s vital signs, the presence of hoarseness, prevention of misaspiration and coughing, the amount and color of neck drainage, and attention to the patient’s perioral, hand and foot numbness and convulsions; (2) observation of PTH and serum calcium (Ca) levels, Between 1.8 and 2.2 mmol/L of serum calcium is the target level, and giving Ca supplementation if necessary treatment: early oral calcium, calcitriol + intravenous calcium infusion at 8-16 g per day; (3) maintenance hemodialysis 1-2 days postoperatively. Patients then received heparin-free hemodialysis one week postoperatively.

### Statistical analysis

The research team employed GraphPad 8.0 for statistical analysis. The team analyzed: (1) measurement data, statistically expressing the data as means ± standard deviations (Mean ± SD), using the independent samples t-test; (2) count data, recorded as percentages, using the chi-square test. *P <*0.05 indicates significant differences.

## Results

### Clinical case data

The research team included 24 SHPT patients in the study and analyzed their data, 13 in the intervention group and 11 in the control group (**Table 1**). No significant differences existed between the groups at age, gender, BMI and kidney disease (*P*>0.05), indicating comparability.

**Table 1:** Comparison of Clinical Data (N=24).

Patient characteristics	EACtPTx+AT Group N=13 Mean ± SD N(%)	OtPTx+AT Group N=11 Mean ± SD N(%)	t/χ2	*P* value
Age	51.54 ± 10.83	57.55 ± 10.78	1.357	0.189
Gender				
Male	7(53.8)	2(18.18)	3.234	0.072
Female	6(46.2)	9(81.82)		
BMI	21.55 ± 2.93	22.21 ± 4.45	0.441	0.664
Kidney disease				
Yes	13(100)	10(90.9)	1.326	0.249
No	0(0)	1(9.1)		

### Surgical outcomes

**Figure 5:**
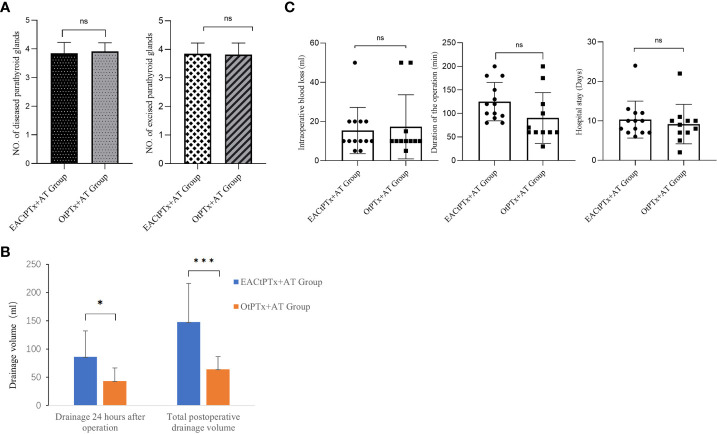
Surgery-related indicators in two groups. **(A)** Comparison of number of diseased and excised parathyroid glands between the two groups. **(B)** Comparison of drainage volume between the two groups. **(C)** Comparison of Duration of the operation. Intraoperative blood loss. Hospital stay between the two groups.

The number of diseased parathyroid glands and excision was 50 in 13 patients in the EACtPTx+AT group and 43 in 11 patients in the OtPTx+AT group, which was a non-significant difference (**Figure 5A**). In the EACtPTx+AT group, the drainage volume of 24 hours after the operation and the total drainage volume were 85ml and 146.2ml, respectively. The drainage volume 24 hours after the operation and the total drainage volume were 45.9ml、68.4ml in the OtPTx+AT group respectively. There were significant differences between the two groups in the 24 hours after the operation and the total drainage volume (**Figure 5B**). The average intraoperative blood loss was 15.4 mL in the EACtPTx+AT group and 17.3 mL in the OtPTx+AT group, which was statistically non-significant (*P* value>0.05) (**Figure 5C**).The average operative time for the EACtPTx+AT group was 125 min compared to 90.45 min for the OtPTx+AT group, which was non-significant (*P* value>0.05) (**Figure 5C**). Average hospital stay was 10.3 days in the EACtPTx+AT group and 9.2 days in the OtPTx+AT group, which was non-significant (*P* value>0.05) (**Figure 5C**). There were no postoperative bleeding, hoarseness or Choking in the two groups, and no conversion to open surgery case in EACtPTx+AT group.

### Clinical efficacy

**Figure 6:**
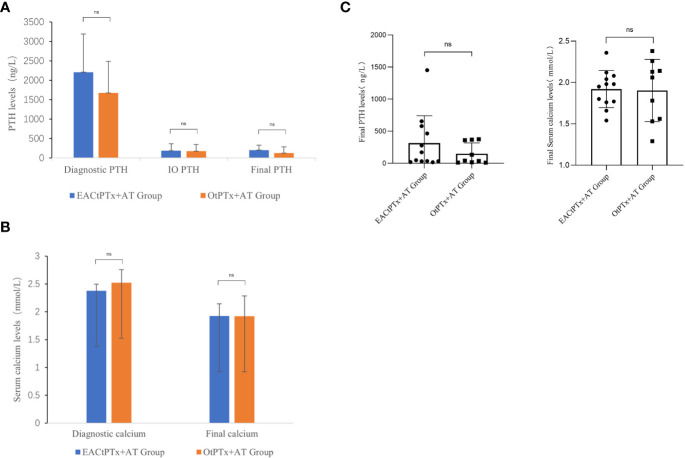
The results of PTH and serum calcium levels in two groups. **(A)** Parathyroid hormone (PTH) levels:Diagnostic PTH, IO PTH, final PTH. **(B)** Serum calcium levels:Diagnostic calcium, final calcium. **(C)** Comparison of postoperative PTH and Serum calcium between the two groups.

In the EACtPTx+AT group, compared with preoperative PTH. Intraoperative and postoperative PTH decreased significantly, showing a significant difference. There were also significant differences in the OtPTx+AT group. In these two groups, postoperative serum calcium decreased compared with preoperative serum calcium, which had a significant difference. Postoperative PTH and postoperative serum calcium decreased significantly in these two groups, and there were no significant differences between the two groups. Diagnostic, intraoperative, and postoperative PTH levels can be seen in **Figure 6A**. Serum calcium levels can be seen in **Figure 6B**. Comparison of postoperative PTH and Serum calcium can be seen in **Figure 6C**. There were no postoperative bleeding, hoarseness or choking in the two groups and no conversion to open surgery case in EACtPTx+AT group.

## Discussion

According to the cross-sectional survey on the prevalence of chronic kidney disease in China, the prevalence of chronic kidney disease in China is 10.8%, and according to the data of the dialysis transplantation registry system, there are about 200,000 maintenance hemodialysis patients in China, and the incidence is gradually increasing, and the number of SHPT patients is also increasing year by year. Because patients with chronic renal failure often have severe cardiovascular and cerebrovascular diseases, osteoporosis, abnormal coagulation mechanisms and severe postoperative hypocalcemia, the perioperative management is difficult and the surgical risk is high, and the clinical treatment of SHPT requires multidisciplinary collaboration. Even if it is carried out, the surgical approach is not standardized, the efficacy is poor, the recurrence rate is high, and the complication rate is high. At present, the diagnosis of early monitoring and early intervention of SHPT caused by chronic renal failure in China needs to be further improved, and patients who are resistant to medical treatment and have indications for surgery should be considered for patients, active surgical treatment should be considered. The surgical treatment process requires multidisciplinary cooperation between the surgical department and nuclear medicine, imaging, ultrasound, endocrinology, anesthesiology, hemodialysis, laboratory, and ICU to develop reasonable and standardized perioperative management in order to obtain the best surgical treatment effect. Our center has made some attempts and explorations to treat secondary hyperparathyroidism with total endoscopic surgery for patients with cosmetic needs. This paper retrospectively analyzed these cases and compared them with open surgery to obtain the retrospective data and endoscopic experience of our center.

Precise preoperative localization plays a crucial role in precise parathyroid surgery and obtaining the best possible surgical outcome. The number and location of the parathyroid glands should be confirmed preoperatively if possible, and in the case of <4 parathyroid glands, any missing glands should be considered and confirmed by selecting the appropriate tests according to the specific situation of the unit. Currently, the common preoperative localization methods used in our hospital include high-frequency ultrasound, CT, and MRI. In this study, high-frequency ultrasound combined with thin-layer CT was used as the main method of preoperative localization and diagnosis. In addition, intraoperative parathyroid ultrasound localization performed by the surgeon is also an important localization method. In our center, intraoperative parathyroid hormone level (IOPTH) is mainly used clinically to help determine the presence of residual ectopic parathyroid glands. However, due to the reduced clearance of iPTH caused by renal insufficiency in SHPT patients and the cross-reactivity of complete and partial PTH, the criteria for IOPTH to determine the success and prognosis of SHPT surgery are still controversial. The results of this study show that of the 13 patients in the endoscopic group, 2 patients had only three parathyroid lesions and the rest had four parathyroid lesions before surgery, while 10 patients in the open group had four parathyroid lesions and 1 patient had only three parathyroid lesions before surgery. Postoperative PTH and serum calcium were significantly decreased in both groups, while those in the endoscopic group were reduced by 80%. In the endoscopy group, one patient underwent complete resection of 4 parathyroid glands, but did not perform intraoperative parathyroid hormone measurement, and found that postoperative parathyroid hormone was not up to the standard. In addition, there were two patients with intraoperative parathyroid hormone reduction that did not reach 80%, and postoperative parathyroid hormone was also not up to the standard. There were two patients in the open group who were lower than 400 ng/L, but the reduction did not reach 80%. We believe that the endoscopic group had a more thorough resection of the parathyroid gland, but the influence of the amount of parathyroid implanted could not be excluded. Therefore, multiple surgeries are required for patients with secondary hyperparathyroidism, as severe nephropathy may accelerate the recurrence of hyperparathyroidism. Patients with progressive renal secondary hyperparathyroidism often cannot fully achieve the desired effect or clinical cure with a single operation. Most patients can only control symptoms and improve their quality of life. This is the confusion of surgical treatment of renal secondary hyperparathyroidism. However, these retrospective clinical data from our center do not affect the safety assessment of endoscopic surgery.

In order to properly treat SHPT, all parathyroid tissue must be resected surgically. In addition to endoscopic tPTx through the anterior chest approach, axilla-bilateral chest approach, and robotic surgery, patients with SHPT have also reported successful outcomes without serious complications (8–12). Our center performed total endoscopic parathyroid surgery through the thoracic approach for some patients with endoscopic requirements and analyzed the data. As for the evaluation of the treatment effect, we can see that postoperative PTH and blood calcium were significantly reduced in the endoscopic group, and there was no significant difference compared with the open group.

In this study, conventional total parathyroidectomy would take around 90.45 min, while the average operation time of EACtPTx+AT group was 125 min, which was comparable with those of other endoscopic approaches. The longer operation time was one of the drawbacks of the endoscopic approach. Average intraoperative blood loss was 15.4 mL in the EACtPTx+AT group and 17.3 mL in the OtPTx+AT group, which was non-significant. The average hospital stay was 10.3 days in the EACtPTx+AT group and 9.2 days in the OtPTx+AT group, which was non-significant. There were no postoperative bleeding, hoarseness or Choking in the two groups, and no conversion to open surgery case in EACtPTx+AT group, concurring with previous studies evaluating the safety of TOEPVA (13–15). In the EACtPTx+AT group, the drainage volume for 24 hours after the operation and the total drainage volume were 85ml and 146.2ml, respectively. The drainage volume for 24 hours after the operation and the total drainage volume were 45.9ml、68.4ml in the OtPTx+AT group respectively. There were significant differences between the two groups in the 24 hours after operation and the total drainage volume. We believe that the increase in drainage volume in endoscopic surgery may be related to the operating space. However, it did not significantly increase the length of hospital stay and did not cause significant discomfort to patients.

Retrospective clinical data in this study showed that the treatment of secondary hyperparathyroidism by total endoscopy through the anterior chest approach was very effective, with fewer postoperative complications and better recovery. On the premise of perfect preoperative preparation, after multidisciplinary discussion, if patients have endoscopic or cosmetic needs, they can choose total endoscopic surgery through the anterior chest approach to treat secondary hyperparathyroidism, and the postoperative efficacy is no less than that of open surgery. In addition, we found that it was more convenient to look for the parathyroid gland under the endoscopic field of view, with less damage to the thyroid gland and easier to protect the surrounding important tissues.

## Conclusion

The risks and significance of Endoscopic parathyroidectomy through anterior chest approach in the treatment of SHPT have not been established, but our present analyses demonstrated that EACtPTx+AT was a good surgical procedure and a highly effective, adequate treatment for SHPT, which we examined achieved superior cosmetic results which clinical symptoms can be significantly relieved, serum PTH and calcium levels can be decreased and did not increase the rate of complications. Patients with parathyroid disease who are concerned about neck scars can elect to undergo endoscopic parathyroidectomy through the anterior chest approach. The detection of intraoperative PTH and the search for pathological parathyroid glands are the key to the success of the operation, more evidence about EACtPTx+AT must be accumulated regarding the effective and safe performance of EACtPTx+AT in the treatment of SHPT and more clinical cases provide data.

## Data availability statement

The original contributions presented in the study are included in the article/**Supplementary material**. Further inquiries can be directed to the corresponding author.

## Ethics statement

The studies involving human participants were reviewed and approved by the Ethics Committee of Ningbo Medical Centre Lihuili Hospital,employee. Written informed consent for participation was not required for this study in accordance with the national legislation and the institutional requirements.

## Author contributions

TH designed the study, TH,JL Collection and analysis of data and wrote the manuscript. JL,YZ Operating and anesthesia team. JY, YL, HZ Chief operating surgeon, provided clinical information and samples. All authors contributed to the article and approved the submitted version.
